# Pharyngeal Airspace Alterations after Using the Mandibular Advancement Device in the Treatment of Obstructive Sleep Apnea Syndrome

**DOI:** 10.3390/life12060835

**Published:** 2022-06-02

**Authors:** Pedro Dias Ferraz, Inês Francisco, Maria Inês Borges, Adriana Guimarães, Fátima Carvalho, Francisco Caramelo, José Pedro Figueiredo, Francisco Vale

**Affiliations:** 1 Coimbra Hospital and University Centre (CHUC), 3000-075 Coimbra, Portugal; 11789@chuc.min-saude.pt (M.I.B.); mfatimacarvalho@live.com.pt (F.C.); jpfigueiredo@fmed.uc.pt (J.P.F.); 2 Faculty of Medicine, Institute of Orthodontics, University of Coimbra, 3000-075 Coimbra, Portugal; ifrancisco@fmed.uc.pt (I.F.); uc45131@uc.pt (A.G.); 3 Area of Environment Genetics and Oncobiology (CIMAGO), Faculty of Medicine, Coimbra Institute for Clinical and Biomedical Research (iCBR), University of Coimbra, 3000-075 Coimbra, Portugal; fcaramelo@fmed.uc.pt; 4 Laboratory of Biostatistics and Medical Informatics (LBIM), Faculty of Medicine, University of Coimbra, 3004-531 Coimbra, Portugal; 5 Centre for Innovative Biomedicine and Biotechnology (CIBB), University of Coimbra, 3000-075 Coimbra, Portugal; 6 Clinical Academic Center of Coimbra (CACC), 3030-370 Coimbra, Portugal

**Keywords:** sleep apnea, obstructive, sleep apnea syndromes, occlusal splints, mandibular advancement devices, sleep disorders, intrinsic, cephalometry

## Abstract

Background: Mandibular Advancement Devices (MADs), inserted in non-surgical treatments for obstructive sleep apnea and hypopnea syndrome (OSAHS), are used intra-orally during the sleep period, with the aim of promoting mandibular protrusion. The aim of the study is to analyze the changes in the upper airway after the use of an MAD in the treatment of OSAHS. Methods: 60 patients diagnosed with OSAHS, as established by the Sleep Medicine Service, underwent treatment with the Silensor SL device at the Stomatology Service of the University Hospital Center of Coimbra, from January 2018 to January 2019. All patients completed two polysomnographies and two lateral teleradiographies: one before starting treatment (T0) and one after 1 year of treatment (T1). In the lateral teleradiography performed after one year of treatment, the patient had the MAD placed intra-orally. The linear measurements of the airspace proposed by the Arnett/Gunson FAB Surgery cephalometric analysis were measured at four craniometric points: A, MCI, B, Pog. Results: The results demonstrate an anteroposterior airway enlargement in two of the four points studied with the MAD placed intra-orally (B and Pog point). The greatest average increase is observed at point Pog (3 mm), followed by B (1 mm), and finally, point A (0.6 mm). Conclusions: This study proved that there is an improvement in anteroposterior measurements at various points in the upper airways after treatment with MAD.

## 1. Introduction

Obstructive sleep apnea and hypopnea syndrome (OSAHS) is a chronic and progressive respiratory disorder characterized by recurrent, total or partial collapse of the upper airways (UA) during sleep. This condition causes cessation of breathing for 10 or more seconds, with changes in the normal sleep pattern and changes in normal pulmonary ventilation, resulting in a deficit of oxygenation [1].

The severity of OSAHS is associated with the number of documented apnea and hypopnea events per hour of sleep (Apnea–Hypopnea Index (AHI)), and, therefore, it can be classified as mild (5 ≤ AHI <15), moderate (15 ≤ AHI < 30) or severe (AHI ≥ 30) [2].

Given the multifactorial characteristics of OSAHS, several possible treatments for its correction are described. Mandibular Advancement Devices (MADs), inserted in non-surgical treatments for OSAHS, are used intra-orally during the sleep period, with the aim of promoting mandibular protrusion. They are seen as a conservative and non-invasive treatment, with high tolerance and adherence to treatment [3,4,5].

It has been described that the use of MADs reduces the collapse of the UA during sleep [6,7]. It is thought that the advancement of maxillary bones leads to an increase in the caliber of the oropharynx and laryngopharynx and provides tension to the muscles involved in this anatomical region. These allow a reduction in UA collapse during deep sleep stages. However, despite more and more studies, the mechanisms that lead to an improvement in MAD are not fully known [7].

OSAHS is usually diagnosed based on physical examination and polysomnographic study (PSG). PSG is currently considered the gold standard method for diagnosing OSAHS and consists of recording chest and abdominal movements during sleep, combined with various parameters, such as AHI, during each hour of sleep. PSG findings are considered normal as long as the AHI is less than one event per hour, with an apnea episode duration of less than 5 s, oxyhemoglobin saturation greater than 90% and less than 10% of the carbon dioxide value at the end of expiration [5].

Profile teleradiography is a standardized and widely available radiographic technique, commonly used in orthodontics to study craniofacial structures. The increase in the dimensions of the UA seems to have an important effect on the success of MAD and, as such, the comparative analysis of craniofacial structures is one of the possible ways to assess the effect of this therapy.

There have been several studies that evaluate the use of cephalometry, which can predict the success of the treatment of OSAHS with an MAD [8,9].

The aim of the study is to evaluate the changes observed in the UA during treatment with the Silensor SL device in previously diagnosed OSAHS.

## 2. Materials and Methods

This study was conducted according to the 1964 Helsinki declaration and its later amendments or comparable ethical standards as well as approved by the Ethics Committee of the Faculty of Medicine at the University of Coimbra (CE-145/2020 on 25 November 2020). All patients gave their written informed consent prior to the start of the study.

First, 60 patients, 38 male and 22 female, with a diagnosis of OSAHS, established by the Sleep Medicine Service, underwent treatment with the Silensor SL device at the Stomatology Service at the Coimbra Hospital and University Centre, from January 2018 to January 2019. The mean age was 52.13 (from 21 to 75 years) and mean body mass index (BMI) was 27.4 kg/m^2^ (from 18.4 to 35.3). Further, 26.6% of patients suffered from obesity (BMI > 30 kg/m^2^). All patients had at least eight teeth in each dental arch, and all presented tolerance to the MAD throughout the entire treatment. Patients with advanced periodontal disease, temporomandibular joint pathology and head and neck malformations syndrome were excluded.

**Figure 1:**
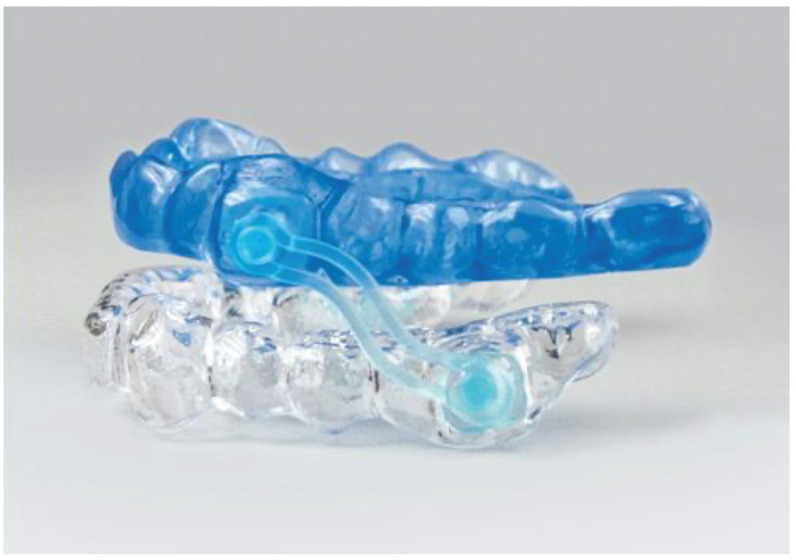
Silensor SL device.

The Silensor SL device consists of two acrylic plates that cover the upper and lower arch, respectively. These two splints are joined by a fixed connector, in the upper part at the level of the canine and in the lower part at the level of the lower first and second molars. This connector orientation allows us to define the mandibular protrusion movement. This device has six connectors of different lengths with the aim of inducing mandibular advancements of 65% to 75% in the maximum protrusion of each patient. This device also allows limited laterality and mouth opening movements [10] (Figure 1).

Patients were classified according to the severity of their disease: mild (AHI of 5–15/h), moderate (AHI of 15–30/h) and severe (AHI > 30/h) [11].

**Figure 2:**
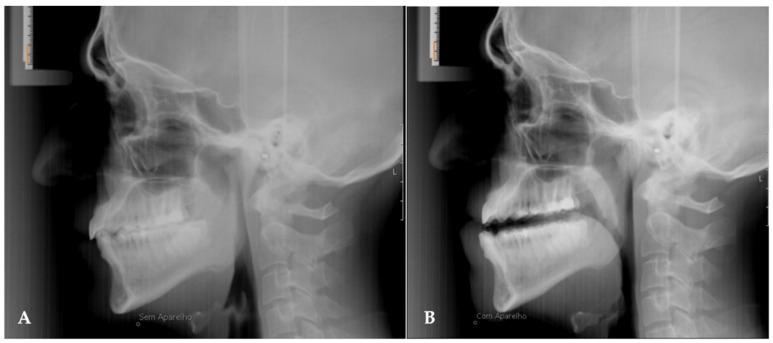
Teleradiography without the device in place (**A**) and teleradiography one year after treatment with the device in place (**B**). The orange line measures 10 mm.

In all patients, two lateral cephalograms were taken, one before starting treatment (T0) and the other after 1 year of treatment (T1). In the final teleradiography (T1) the patients had the MAD placed intra-orally (Figure 2). Moreover, during the evaluation of the results, all patients underwent cardiorespiratory study polysomnography (AASM level III).

To carry out this study, cephalometric analysis was performed using the Dolphin Image software, version 11.9 (DolphinImage & Management Solutions^®®^, Chatsworth, CA, USA), using the Arnett/Gunson FAB Surgery method. To transfer the acquired images to the virtual environment, the spatial orientation was previously obtained according to the Frankfurt Horizontal Plan (FHP).

The actual movement presented by each patient after the placement of the MAD was quantified at the Pogonion point (Pog), after the superimposition of the pre- and post-treatment radiographs of each patient. The superimposition method adopted was based on “The structural method” developed by Arne Björk [10,12].

**Figure 3:**
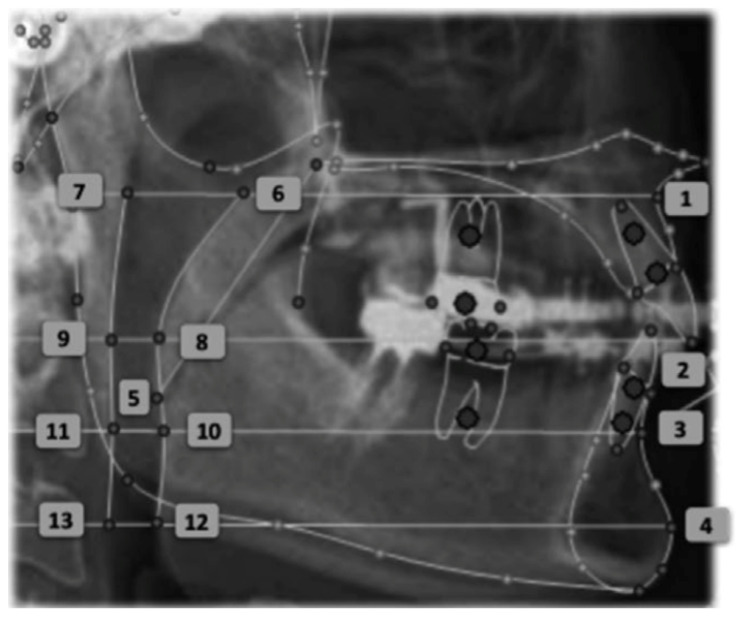
Cephalometric points: (1) A-point, (2) MCI-point, (3) B-point, (4) Pog-point, (5) A/G tip of soft Palate, (6) A/G anterior SPAS at A, (7) A/G posterior SPAS at A, (8) A/G anterior SPAS at MCI, (9) A/G posterior SPAS at MCI, (10) A/G anterior SPAS at B, (11) A/G posterior SPAS at B, (12) A/G anterior SPAS at Pog, (13) A/G posterior SPAS at Pog.

The linear measurements of the airspace proposed by the Arnett/Gunson FAB Surgery cephalometric analysis were measured at four craniometric points: A, MCI, B, Pog (Figure 3):

- SPAS at point A (SPAS at A): A line is drawn perpendicular to the true vertical line that passes through point A and extends posteriorly, intersecting the anterior (A/G anterior SPAS at A) and posterior (A/G posterior SPAS at A) limits of the superior posterior airway space. The dimension of the UA is given by the distance between these two points.
- SPAS at point MCI (SPAS at MCI): A line is drawn perpendicular to the true vertical line that passes through point MCI (point located on the incisal edge of the maxillary central incisor) and extends posteriorly, intersecting the anterior (A/G anterior SPAS at MCI) and posterior (A/G posterior SPAS at MCI) limits of the superior posterior airway (Figure 3—Points 8 and 9).
- SPAS at point B (SPAS at B): A line is drawn perpendicular to the true vertical line that passes through point B and extends posteriorly, intersecting the anterior (A/G anterior SPAS at B) and posterior (A/G posterior SPAS at B) limits of the superior posterior airway space.
- SPAS at point Pog (SPAS at Pog): A line is drawn perpendicular to the true vertical line that passes through point Pog at the anterior (A/G anterior SPAS at Pog) and posterior (A/G posterior SPAS at Pog) wall of the superior posterior airway.

A power analysis was performed for the variables studied using the G*Power 3.1.9.7 software and the bilateral Student’s t-test. The observed potency was 99.9% for Pog point, 74.5% for B point, 99.9% for SNB, and 99.9% for ANB. The variables under study were described as mean, standard deviation, maximum and minimum. All statistical tests were performed after evaluating the normality of distribution of quantitative variables with the Shapiro–Wilk test. To assess the difference between the changes observed in the AHI index and oxygen saturation after MAD treatment the Wilcoxon test was performed. BMI difference after MAD treatment was analyzed using the Student’s t-test. Spearman correlation was performed in order to verify whether the variation in BMI could explain the variation observed in the AHI. Statistical analysis was performed on the IBM^®®^ SPSS^®®^ v26 platform, adopting a significance level of 0.05.

## 3. Results

The sample selected consisted of 60 individuals (38 males and 22 females) with an average of 52.1 (SD = 9.92). The distribution of patients according to AHI classification before and after MAD treatment is presented in Table 1. All patients showed an improvement in the AHI index. Patients with severe AHI in T0 achieved a moderate AHI after MAD treatment. Only one patient with a moderate AHI index maintained the severity, however, with improvements in the AHI index (21 to 17). Table 2 presents the results of the BMI, AHI and oxygen saturation changes.

Regarding the AHI index, an improvement was found with statistically significant differences after MAD treatment (*p* < 0.001). Comparisons between TO and T1 showed no significant differences for SaO_2_ and BMI variables after MAD treatment (*p* = 0.754 and *p* = 0.550, respectively).

**Figure 4:**
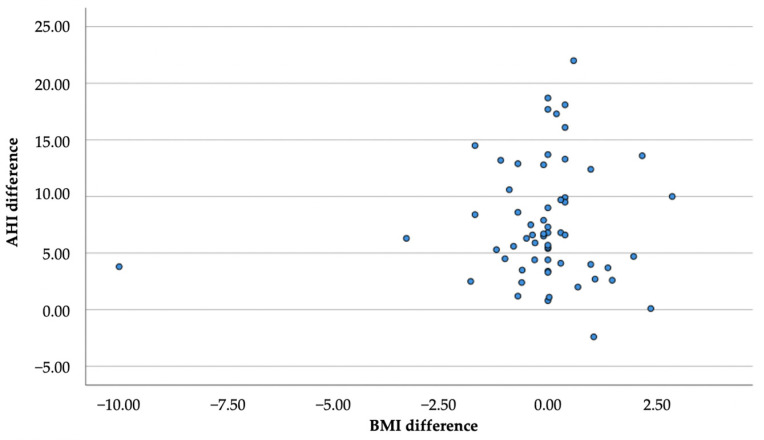
Correlation between AHI and BMI.

There was no statistically significant correlation (r = 0.000; *p* = 0.998) between the difference in BMI (TO and T1) and the difference in AHI (TO and T1), which shows that the two variables are independent of each other (Figure 4).

Table 3 shows the results of changes in the posterior UA: an anteroposterior increase was observed with the MAD placed intra-orally. The greatest average increase was observed at point Pog, followed by B, and finally point A.

Regarding point A, there was a slight advance from an average value of 14.67 mm to 15.27 mm. In B, the anteroposterior advancement was 1 mm from 10.89 mm to 11.88 mm. The biggest difference was found at the Pog point level (practically 3 mm from 12.92 mm to 15.72 mm). At the MCI point, there were no significant changes (Table 3).

**Figure 5:**
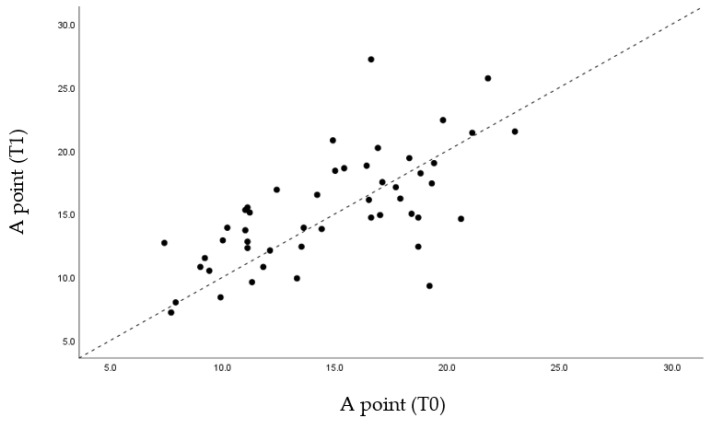
Dispersion of measurements before and after using the MAD at point A.

**Figure 6:**
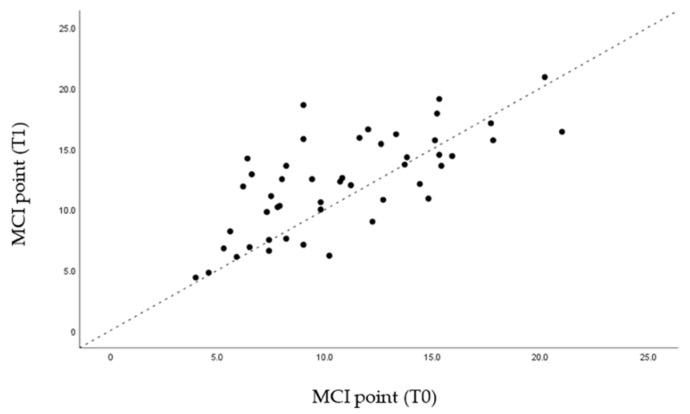
Dispersion of measurements before and after using the MAD at point MCI.

**Figure 7:**
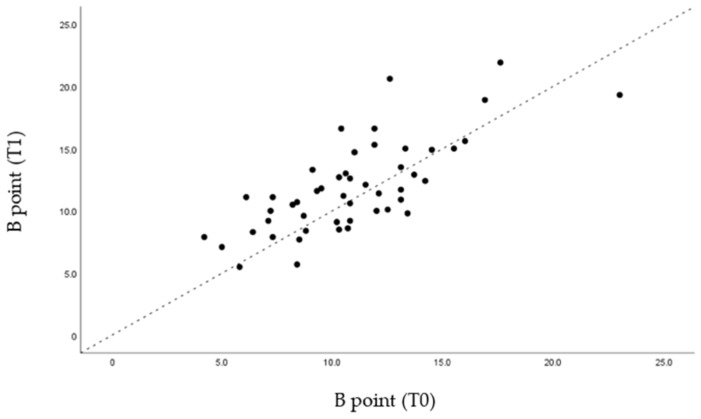
Dispersion of measurements before and after using the MAD at point B.

**Figure 8:**
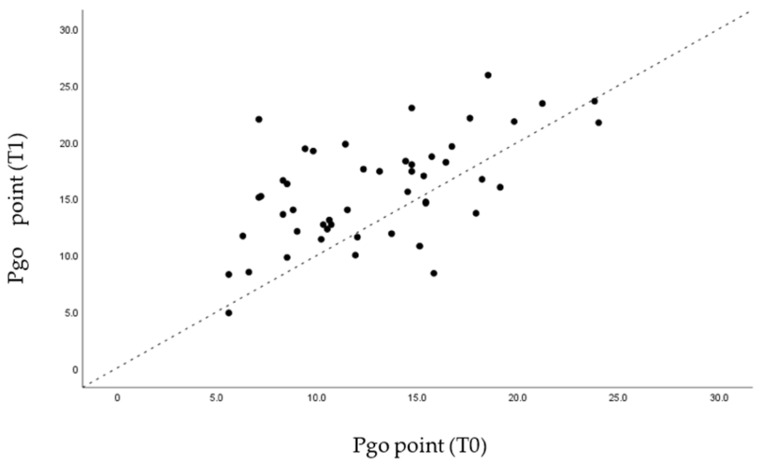
Dispersion of measurements before and after using the MAD at point Pog.

Through the graphs presented (Figure 5, Figure 6, Figure 7 and Figure 8), it is possible to interpret the individual variation in each patient. Dispersion diagrams of the measurements before and after use of the device are presented. In each of the diagrams, the line y = x is shown so that one can compare visually if the values increase (the points are above the line) or decrease (the points are below the line).

**Figure 9:**
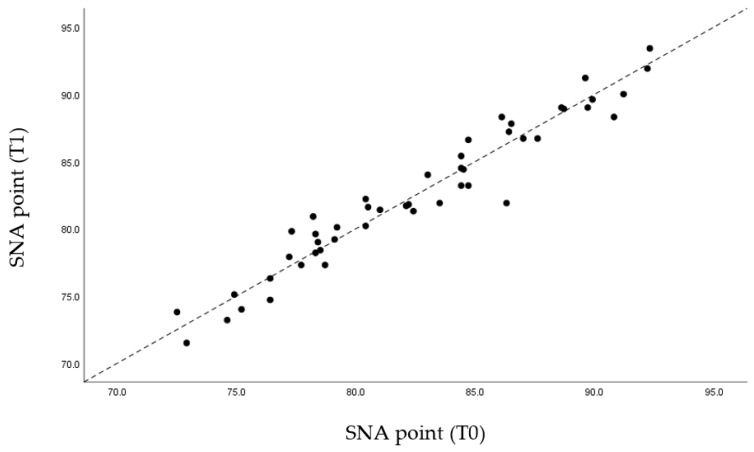
Dispersion of measurements before and after using the MAD with SNA angle.

**Figure 10:**
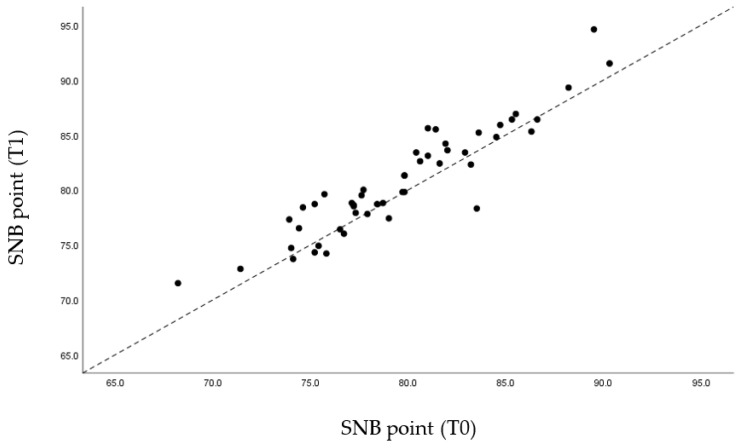
Dispersion of measurements before and after using the MAD with SNB angle.

Regarding the skeletal reference points, the SNA values remained unchanged due to the fact that there was no movement of the jaw in the MAD mechanism (Figure 9). Due to the mandibular advance caused by the MAD, as expected, the SNB values rose from an average of 79.64 mm to 80.77 mm (Figure 10). Likewise, the value of the angle formed by ANB decreased slightly from 2.77° to 1.69°, also due to the mandibular advance caused by MAD (Table 3).

In relation to the skeletal analysis, it was found that, according to the SNA angle, the values were very close to the original values. The fact that MAD causes the advance of the mandible results in point A remaining in the same position before and after treatment (Figure 5).

Regarding the SNB angle, the results shown in Figure 10 are predictable, due to the mandibular advancement caused by the MAD, which provoke the advancement of point B in space.

As for the ANB angle, the vast majority of the population studied presents an angle variation in negative values also due to mandibular advancement.

## 4. Discussion

MAD allow the mandible to be repositioned and stay stable with a minimal mouth opening, so that it keeps the tongue against the floor of the oral cavity, contributing to an increase in the caliber of the UA [13,14]. The sagittal pharyngeal cross-sectional area increased after the application of MAD. The results confirm the mechanical effectiveness of the Silensor SL appliance.

Through statistical analysis, we verified that in all the graphs presented (Figure 5, Figure 6, Figure 7 and Figure 8), most of the points representing each patient studied are above the x = y line, which generally confirms the increase in the sagittal dimensions of the airway.

Most of the published studies emphasize that the main mechanism of action of the MAD consists of the mechanical advancement of the mandible and the increase in the anteroposterior dimensions of the oropharynx, thus, avoiding the collapse of the UA during sleep [6,15]. Other studies report that mandibular advancement improves the caliber of the UA, and that it occurs predominantly both due to the increased volume of the velopharynx and due to the increase in its lateral dimensions [16].

A possible mechanism leading to the widening of the airways in the palatal plane is related to the tension transmitted along the palatoglossus muscles to the soft palate. As the soft palate advances, tension is transmitted along the palatopharyngeal muscle to the posterior pharyngeal wall. This tension on the posterior pharyngeal wall increases the lateral volume of part of the oropharynx [17].

Other studies demonstrate that, with the placement of the MAD, the total area of the sagittal cross-section of the tongue significantly increased, which means that the shape and posture of the tongue change after insertion of MAD [16]. The sagittal sectional area of the tongue is enlarged and positioned inferiorly in the supine position. The explanation found is related to gravitational attraction and the mechanical effectiveness of the device [13].

This study presents an evaluation of the effectiveness of MADs through a widely used method for the study of UA. On the other hand, it is a valuable sample compared to existing studies. The constant and rigorous method was used when performing a profile teleradiography before and after a year of treatment [14]. Finally, the results of this study should make clinicians aware of the need to examine the oral cavity since it can be increasingly involved in the pathophysiology of OSAHS, so they can provide a valuable contribution to the screening of this pathology.

Nonetheless, this study also included some difficulties and limitations. It is not always easy to identify all the cephalometric points, due to distortions in the radiographic image or to the overlapping of structures, as this is a region with several anatomical structures involved, and, in fact, more than one image exam is needed to assess the dimensions of the airway. Although cephalometry can be considered an important tool for performing comparative skeletal measurements, it may have its known limitations. The lateral teleradiographies represent a bi-dimensional image of a three-dimensional structure, which makes its accuracy controversial. The alternative to the method used for cephalometric assessment would be cone beam computed tomography. However, the potential benefits of diagnosis and treatment planning do not outweigh the potential risks of an increase in radiation dose [10,18]. Moreover, further studies should be carried out with larger samples and follow-up.

## 5. Conclusions

Obstructive sleep apnea and hypopnea syndrome is considered a public health problem due to its high prevalence, being responsible for several short- and long-term co-morbidities. This study proved that there is improvement in anteroposterior measurements, with statistically significant values, at various points in the UA, namely in the treatment of mild to moderate OSAHS.

## Figures and Tables

**Table 1:** Distribution of patients according to AHI classification and sex.

	AHI	Total	Female	Male
T0	Mild	39	12	27
	Moderate	19	10	9
	Severe	2	0	2
T1	Mild	57	21	36
	Moderate	3	1	2
	Severe	0	0	0

**Table 2:** Descriptive statistics for the measured variables.

	Variables	Mean	SD	Minimum	Maximum
T0	BMI	27.40	3.32	18.30	35.30
	AHI	14.10	6.01	5.20	34.00
	SaO_2_ (%)	94.30	1.78	84.90	98.00
T1	BMI	27.50	3.43	18.40	39.10
	AHI	6.50	5.46	0.00	27.90
	SaO_2_ (%)	94.40	2.20	86.00	98.00

**Table 3:** Cephalometric measurements.

Variable	T0	T1	Difference	*p* ^§^
A	14.67 ± 4.16 (7.40/23.00)	15.27 ± 4.39 (7.20/27.20)	0.59 ± 3.48 (−9.90/10.60)	0.238
MCI	12.40 ± 11.77 (4.00/88.00)	12.06 ± 4.03 (4.40/20.90)	−0.34 ± 12.06 (−80.30/9.60)	0.844
B	10.89 ± 3.51 (4.20/23.00)	11.88 ± 3.66 (5.50/21.90)	0.99 ± 2.60 (−3.70/8.00)	0.010
Pog	12.92 ± 4.67 (5.60/24.00)	15.72 ± 4.64 (4.90/25.90)	2.80 ± 4.24 (−7.40/14.90)	<0.001
SNA	82.48 ± 5.31 (72.50/92.30)	82.48 ± 5.37 (71.50/93.40)	0.00 ± 1.35 (−4.40/2.70)	0.992
SNB	79.64 ± 4.70 (68.20/90.30)	80.77 ± 4.91 (71.50/94.60)	1.13 ± 1.85 (−5.20/5.10)	<0.001
ANB	2.77 ± 2.49 (−2.30/8.80)	1.69 ± 2.90 (−4.70/7.00)	−1.07 ± 1.58 (−4.80/2.00)	<0.001

^§^ Student’s *t*-test.

## Data Availability

The data presented in this study are available on request from the corresponding author.
